# Protocol for identifying immune mediators during *Leishmania* infection in mice using metabolomic analysis

**DOI:** 10.1016/j.xpro.2025.103817

**Published:** 2025-07-16

**Authors:** Thalia Pacheco-Fernandez, Laura Klenow, Nazli Azodi, Hannah Markle, Matthew Bernier, Greta Volpedo, Timur Oljuskin, Parna Bhattacharya, Shinjiro Hamano, Greg Matlashewski, Hira L. Nakhasi, Abhay R. Satoskar, Sreenivas Gannavaram

**Affiliations:** 1 Division of Emerging and Transfusion Transmitted Diseases, Office of Blood Research and Review, Center of Biologics Research and Evaluation, Food and Drug Administration, Silver Spring, MD 20993, USA; 2 The Ohio State University, Columbus, OH 43201, USA; 3 Department of Neurosciences, Rehabilitation, Ophthalmology, Genetics, Maternal and Child Health, University of Genoa, 16126 Genoa, Italy; 4 Animal Parasitic Diseases Laboratory, Agricultural Research Service, USDA, Beltsville, MD 20705, USA; 5 Center for Devices and Radiological Health, Food and Drug Administration, Silver Spring, MD 20993, USA; 6 Department of Parasitology, Institute of Tropical Medicine (NEKKEN), The Joint Usage/Research Center on Tropical Disease, Nagasaki University, Sakamoto, Nagasaki 852-8523, Japan; 7 Department of Microbiology and Immunology, McGill University, Montreal, QC H3A2B4, Canada

**Keywords:** metabolomics, microbiology, model organisms, molecular biology, mass spectrometry

## Abstract

Here, we present a protocol for identifying immune mediators during *Leishmania* infection in mice using metabolomic analysis. We describe steps for *Leishmania* infection, harvesting infected tissues, and performing liquid chromatography-mass spectrometry (LC-MS) to identify enriched metabolic pathways and altered metabolites. We then detail procedures for verifying the metabolite role in immune response via PCR and ELISA. This protocol focuses on cutaneous leishmaniasis models, but the analyses here are applicable to other infection models.

For complete details on the use and execution of this protocol, please refer to Volpedo et al.^1^ and Oljuskin et al.^2^

## Highlights

• Development of a murine model of infection with two dermotropic *Leishmania* species
• Metabolite extraction from infection site and downstream characterization via HPLC
• Metabolic pathway analysis using MetaboAnalyst and MetScape
• *In vitro* validation via orthogonal methods, such as PCR and ELISA

## Abstract (editor highlights)

Publisher’s note: Undertaking any experimental protocol requires adherence to local institutional guidelines for laboratory safety and ethics.

## Graphical abstract

**Figure:**
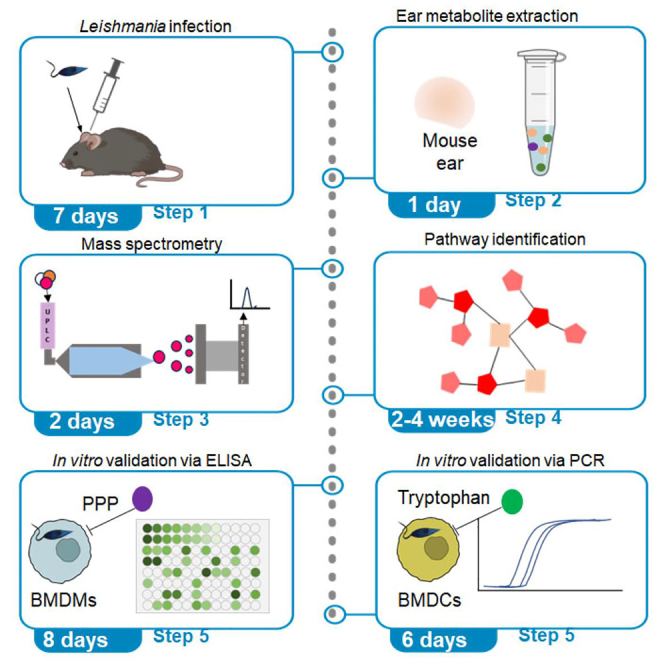

## Before you begin

This protocol combines the methods used in two different studies on *Leishmania mexicana* and *Leishmania major* infections in murine models. The protocol below describes the specific steps for untargeted metabolomics in pre-clinical models of cutaneous leishmaniasis using *Leishmania (L.) mexicana* and *Leishmania (L.) major* parasites, both the wild type (WT) and the c*entrin* knock out (*Cen*^−/−^) strains. However, the protocol can be applied to infections with other strains of *Leishmania* or other protozoan parasites by modifying the tissue sample and the animal model accordingly. Prior to the study, proper animal protocol approvals must be acquired, virulent parasites should be generated through passaging in susceptible animal models, and a well-growing stock of promastigotes should be cryogenically banked to maintain experimental consistency. Additionally, we recommend the validation of the results with appropriate experiments, such as in this protocol we describe the use of chemical inhibitors of the pathways that control cytokine levels to detect changes in cytokine profile of infected phagocytes *in vitro*.

### Institutional permissions

Animal experiments were performed at two locations following the approval of animal protocols and in accordance with all Institutional Animal Care and Use guidelines (IACUC): 1) The Ohio State University animal facility (protocol #2010A0048-R3); and 2) The Food and Drug Administration (FDA) (protocol #1995-26). Additional time will be required for the institutional review and approval of all protocols by your organization’s committee.

### Generating virulent parasite stocks

**Timing: 2–3 weeks**

Passing *Leishmania* parasites through immunocompetent mice is critical to maintain virulence characteristics. Culturing parasites recovered from infected hosts will generate stocks that can be used for experimental infections. Conditions described in this section were the same for the WT and *Cen*^−/−^ parasites from each species.

1. Select the genetic background for resistant mice depending on the strain of *Leishmania.*
2. Identify the method of inoculation based on the *Leishmania* strain and the draining lymph node to harvest. In our case we used the following:

**Table:** 

*Leishmania* strain	Mouse strain	Site of inoculation	Route of inoculation	Draining lymph node
*L. major* Friedlin (FV9)	BALB/c (6-8 weeks of age)	Footpads	Intradermal	Superficial cervical
*L. mexicana* (MNYC/B2/62/m379)	129S6/SvEvTac (6-8 weeks of age)	Back rumps	Subcutaneous	Inguinal

3. Inject 5 × 10^6^ parasites per mouse.
4. 7-14 days post infection, collect the draining lymph nodes and place them in a petri dish containing 3 mL of complete M199 (see recipe in materials and equipment section).
5. Macerate the lymph nodes against a 40 μm strainer using the back of a syringe plunger.
6. Transfer 200-500 μL of the cell homogenate to a T-25 vented flask with 10 mL of complete M199 medium.
7. Culture the parasites at 26-27°C for 7-14 days until promastigotes are seen in the culture.
8. Stationary phase promastigotes obtained from serial passages 3-5 are ideal for infection experiments. Cryopreserve remaining parasites for further experiments.
9. Cryopreserve parasites by pelleting the culture (centrifuge at 3000 x g for 10 min) and resuspending in a glycerol freezing solution.
  a. To make the freezing solution, autoclave 75% glycerol.
  b. Add 1 mL of 75% glycerol to 9 mL of parasite-specific medium for every 10 mL of parasite culture.
  c. Resuspend the pelleted parasites in the freezing solution and store in Nunc cryotubes in a freezing container at −80°C, overnight (18-20 h). The following day, transfer samples to long term liquid nitrogen storage in a cryobox.

**Pause Point:** Cultures can be kept frozen in liquid nitrogen for many years (over 5 years).

### Maintaining parasite cultures

**Timing: 30 min, 2 times a week**

Passage parasites every 3-4 days depending on the culture density to maintain actively dividing stocks. Do not exceed 3-5 passages total.

**Figure 1:**
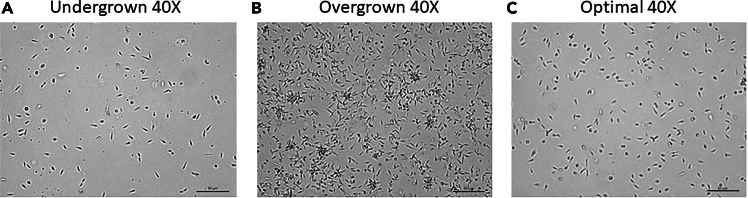
Examples of *Leishmania* cultures growth Microscopic pictures taken at 40X of a live viable *Leishmania* culture show an **A)** undergrown parasite culture (∼10% confluent), and **B)** an overgrown culture of parasites (∼90% confluent). Finally, **C)** a culture in optimal density for use in infection (∼60% confluent) at 40X magnification. Scale bars represent 50 μm.
11. Once the culture reaches high density (30-40 × 10^6^/mL), pass the parasites into a new flask.

***Note:*** High density is marked by expansion of active promastigotes; some may elongate into the metacyclic phase and will be actively mobile. Infrequent rosettes may have formed, indicating healthy, densely populated flasks.

***Note:*** Do not let the flasks become overly dense (Figure 1) which can also be seen by an evident change of the color of the media from red to orange.

10. After obtaining a promastigote culture, check daily for parasite density (Figure 1).

12. Add 12-26 mL of media to a vented, T-75 flask (depending on total culture volume needed).
13. Add 2-4 mL of the current parasite culture and gently mix by tilting the flask side to side.
14. Incubate the prepared flask at 26-27°C and monitor, until the next passage is needed.
15. At this stage, the parasites are ready to be used for infection.

***Note:*** For *in vivo* infections, confirm parasites are in the metacyclic stage as these parasites are the most infectious. Metacyclic parasites can be visually identified by their slender and elongated appearance, as well as highly active flagella. In contrast, procyclic promastigotes will appear shorter and broader, with shorter flagella and less motility.

## Key resources table

**Table:** 

REAGENT or RESOURCE	SOURCE	IDENTIFIER
**Chemicals, peptides, and recombinant proteins**		

Liberase TL	Roche	Cat. No. 5401020001
DL-Kynurenine	Sigma-Aldrich	Cat. No. 61250
1-Methyl-D-Tryptophan	Tocris	Cat. No. 5698
Melatonin	Sigma-Aldrich	Cat. No. M5250-1G
4-Chloro-DL-Phenylalanine (4-CDP)	Sigma-Aldrich	Cat. No. C6506-5G
L-10 TaqMan gene expression assay	Thermo Fisher Scientific	Mm00439614_m1
AhR TaqMan gene expression assay	Thermo Fisher Scientific	Mm00478932_m1
IDO-1 TaqMan gene expression assay	Thermo Fisher Scientific	Mm00492590_m1
*inf-g* TaqMan gene expression assay	Thermo Fisher Scientific	Mm01168134_m1
*Il-12b* TaqMan gene expression assay	Thermo Fisher Scientific	Mm00434174_m1
*tnf-a* TaqMan gene expression assay	Thermo Fisher Scientific	Mm00443258_m1
*gapdh* TaqMan gene expression assay	Thermo Fisher Scientific	Mm99999915_g1
FICZ	Sigma-Aldrich	Cat. No. SML1489
IL-4	ProSpec	Cat. No. cyt-282-b
GM-CSF	ProSpec	Cat. No. cyt-222-b
Gentamicin (10 mg/mL)	Gibco	Ref No. 15710-064
DMEM	Gibco	Cat. No. 11965092
RPMI	Gibco	Cat. No. 11875093
Fetal bovine serum	VWR	Cat. No. 89510-196
Penicillin/streptomycin	Gibco	Cat. No. 15140122
6-biopterin	Sigma-Aldrich	Cat. No. B2517
M199	Gibco	Cat. No. 11150067
Avidin D-HRP	Vector Laboratories	Cat. No. ZK0224
1-Step TMB ELISA substrate solutions	Thermo Fisher Scientific	Cat. No. 34021
Capture anti-IL-12 clone C18.2	BioLegend	Cat. No. 511802 (RRID: AB_2123769)
Capture anti-IL-1β clone B122	BioLegend	Cat. No. 503502 (RRID: AB_315287)

**Critical commercial assays**		

Applied Biosystems high-capacity cDNA reverse-transcription kit	Thermo Fisher Scientific	Ref. No. 4368814
TaqMan gene expression master mix	Thermo Fisher Scientific	Ref. No. 4369016
Invitrogen PureLink RNA mini kit	Thermo Fisher Scientific	Ref. No. 12183018A

**Experimental models: Organisms/strains**		

Leishmania major Cen^−/−^	Zhang et al.^3^	LmCen^−/−^ derived from the parental L. major Friedlin (FV9) strain
Leishmania major WT	N/A	L. major Friedlin (FV9)
Mouse: BALB/c (female 6–8 weeks old)	The Jackson Laboratory	RRID: IMSR_JAX:000651
Mouse: 129S6/SvEvTac (female, 6–8 weeks old)	The Jackson Laboratory	RRID: IMSR_JAX:021315
Mouse: C57BL/6J (female, 4–6 weeks old)	The Jackson Laboratory	RRID: IMSR_JAX:000664

**Software and algorithms**		

MetaboAnalyst 4.0	Xia et al.^4^	MetaboAnalyst; RRID: SCR_015539
MetScape 3.1.3	Karnovsky et al.^5^	MetScape 3.1.3 (ncibi.org); RRID: SCR_014687
Kyoto Encyclopedia of Genetics and Genomes (KEGG) COMPOUND	Kanehisa et al.^6^	KEGG: Kyoto Encyclopedia of Genes and Genomes; RRID: SCR_001120
GraphPad Prism 9	Dotmatics	Home – GraphPad; RRID: SCR_002798
Ingenuity Pathway Analysis (IPA) Version 111725566	Kramer et al.^7^	Ingenuity Pathway Analysis | QIAGEN Digital Insights; RRID: SCR_008653
The Chemical Translation Service (CTS) 3.4.0.	Wohlgemuth et al.^8^	CTS Proxy version 3.4.0 (https://cts.fiehnlab.ucdavis.edu/services) RRID:SCR_014681

**Other**		

35 micron sterile Medicon	BD Biosciences	Cat No. 340589
Filcon, sterile, syringe-type	BD Biosciences	Cat No. 340606
18G × 1-1/2 in BD PrecisionGlide needle	BD Biosciences	Cat. No. 305196
25G × 5/8 in BD PrecisionGlide needle	BD Biosciences	Cat No. 305122
Cellometer Vision cell profiler	Nexcelom Bioscience	Item: 26775
Disposable cell counting chamber	Nexcelom Bioscience	Cat No. CHT4-PD100-002
Medimachine	Becton Dickinson	Cat No. 340587
Medicons	Becton Dickinson	Cat No. 340589
LTQ XL linear ion trap mass spectrometer	Thermo Fisher Scientific	Cat. No. IQLAAEGAAVFACZMAIK
Bio-Rad C1000 touch thermal cycle CFX96 real-time system	Bio-Rad	Cat. No. 1845097
Biometra TOne 96G, 230 V	Analytik Jena	Cat. No. 846-2-070-301
SpectraMax M3 multi-mode microplate reader	Molecular Devices	Cat. No. 89429-536
Cell culture incubator (26°C–27°C), no CO_2_ injection	Thermo Scientific	Cat No. PR205745R
Cell culture incubator (37°C) with 5% CO_2_ injection	Sanyo Scientific	Cat No. MCO-18AIC
Centrifuge (up to RCF of 13,500 × *g*)	Thermo Scientific	Cat No. TSO-LEGXTR
ViaStain AOPI staining solution	Nexcelom Bioscience	Cat No. CS2-0106
5 mL serological pipet	Fisherbrand	Cat No. 13-678-11D
10 mL serological pipet (Fisherbrand)	Fisherbrand	Cat No. 13-678-11E
25 mL serological pipet	Fisherbrand	Cat No. 13-678-11
3 mL syringes	BD Biosciences	Cat No. 309656
10 mL syringes	BD Biosciences	Cat No. 302995
Insulin syringes	BD Biosciences	Cat No. 329461
18-gauge needle	BD Biosciences	Cat No. 305196
25-gauge needle	BD Biosciences	Cat No. 305122
Scalpel	Bard Parker	Cat No. 372620#20
6-well plates	Falcon	Cat No. 353046
Cell scraper	Fisherbrand	Cat No. 08-100-241
96-well flat bottom plates	Falcon	Cat No. 353072
Parafilm	Bemis	Cat No. PM-996
25 mL sterile reservoirs	Corning	Cat No. 4870
Hard shell PCR plates	Bio-Rad	Cat No.HSP9641
Microseal ‘B’ seal	Bio-Rad	Cat No.MSB1001
RBC lysis buffer (10X)	BioLegend	Cat. No. 420301
15 mL conical tubes	Falcon	Cat No. 352096
50 mL conical tubes	Falcon	Cat No. 352070
1.5 mL Eppendorf tubes	Fisherbrand	Cat No. 05-408-129
0.2 mL PCR tubes	USA Scientific	Cat No.1402-8100

## Materials and equipment

This section provides a list of other materials and reagents/media that will be needed by the researcher to perform the experiment.

**Table:** *L. major* media

Reagent	Final concentration	Amount
M199 powder	1X	5.34 g
Heat- inactivated fetal bovine serum	20%	100 mL
1 M HEPES buffer	210 μM	20 mL
Penicillin/Streptomycin	1X	5 mL
Adenine	0.1 mM	5 mL
Hemin	0.0005%	5 mL
Biotin	0.00005%	250 μL
ddH2O	N/A	∼365 mL
**Total**	**N/A**	**500 mL**

Note on storage conditions: Adjust pH to 7.0. Store at 4°C for up to 3 months

**Table:** *L. mexicana* media

Reagent	Final concentration	Amount
M199 media	N/A	500 mL
Heat- inactivated fetal bovine serum	10%	50 mL
1 M HEPES buffer	839 μM	5 mL
Penicillin/Streptomycin	1X	5 mL
ddH2O	N/A	∼365 mL
**Total**	**N/A**	**500 mL**

Note on storage conditions: Adjust pH to 7.2. Store at 4°C for up to 3 months

**Table:** Supplemented media for parasite burden

Reagent	Final concentration	Amount
Species specific media	N/A	100 mL
Gentamicin	0.01 mg/mL	100 μL
6-biopterin	0.002 μg/mL	1 mL
**Total**	**N/A**	**101.1 mL**

Note on storage conditions: Store at 4°C for up to 3 months

**Table:** Liberase solution per ear

Reagent	Final concentration	Amount
Liberase	0.2 mg/mL	0.6 mg
DMEM	1X	3 mL
Penicillin/Streptomycin	1X	300 μl
**Total**	**N/A**	**3 mL**

Note on storage conditions: Make this solution the day it is needed and maintain on ice

### Complete RPMI

Remove 50 mL of RPMI prior to the addition of supplemental reagents.

**Table:** 

Reagent	Final concentration	Amount
Heat inactivated FBS	10% vol/vol	50 mL
Penicillin/Streptomycin	1X	5 mL
L-Glutamine	1X	5 mL
**Total**	**N/A**	**510 mL**

Note on storage conditions: Store at 4°C for up to 3 months

**Table:** Adenine stock solution

Reagent	Final concentration	Amount
HEPES (1 M), pH 7.5–8.0	50 mM	5 mL
Adenine	10 mM	171.6 mg
H_2_O	N/A	95 mL
**Total**	**N/A**	**100 mL**

Note on storage conditions: Store at −20°C for up to 6 months

**Table:** Hemin stock solution

Reagent	Final concentration	Amount
Hemin	0.25%	125 mg
Triethanolamine	50%	25 mL
H_2_O	N/A	25 mL
**Total**	**N/A**	**50 mL**

Note on storage conditions: Store at −20°C for up to 6 months

**Table:** Biotin stock solution

Reagent	Final concentration	Amount
Biotin	0.1%	50 mg
Ethanol	95%	50 mL
**Total**	**N/A**	**50 mL**

Note on storage conditions: Store at −20 °C for up to 6 months

## Step-by-step method details

This section lists the major steps to infect mice, obtain samples and perform metabolomic analysis. Here we also included two validation methods.

### Mouse parasite injections

**Timing: 1 h**

Here we describe the procedure used for parasite injection of *L. major* WT, *L. major Cen*^−/−^*, L. mexicana* WT, and *L. mexicana Cen*^−/−^*.* The same method of injection can be followed for different *Leishmania* species.

1. Verify that the parasites are ready for injection (in stationary phase) as described above (section describing “Generating virulent parasite stocks”).
2. Count 1 × 10^6^ parasites per mouse and resuspend in 10 μL PBS.

**CRITICAL:** Always prepare a greater volume of parasite suspension accounting for 2-3 extra injections.

3. Inject the parasites intradermally in the ear dermis of 6–8-week-old female C57Bl/6 mice.
  ***Note:*** The mouse genetic background, age and strain will defer depending on the infection model used.
  a. Fully anesthetize mice with isoflurane according to your approved protocols.
  b. Inject 10 μL of the prepared parasite suspension with a 31-gauge insulin syringe with a 5/16-inch needle.
    ***Optional:*** When injecting, wrap the ear around the base of a pen or the bottom of an Eppendorf tube to ensure a flat surface and avoid folding the ear during injection. Use a piece of clear, double-sided tape to secure the ear to the pen or tube. Using this method avoids placing your finger inside the ear that helps to prevent accidental needle stick injuries (Figure 2).
    **Figure 2:**
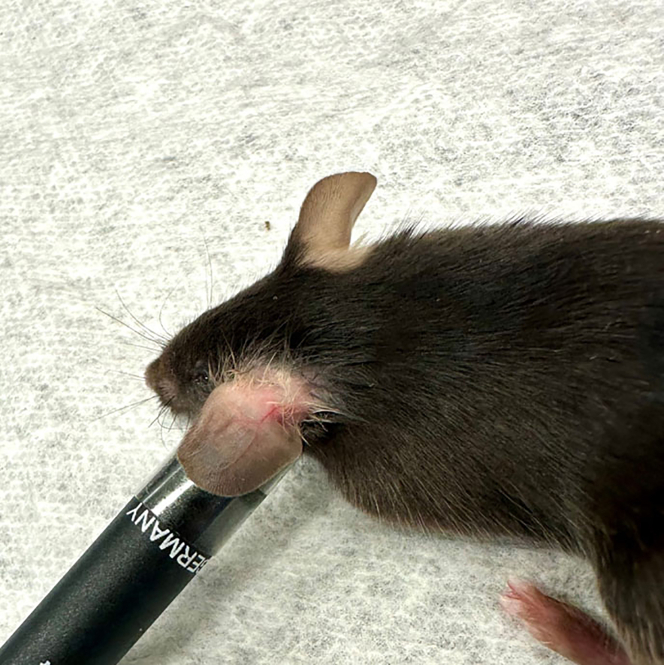
Securing mouse ear for intradermal inoculation Depiction of how to wrap the ear around the back of a pen using double sided tape to avoid accidental finger injection.

### Animal tissue harvesting and processing: Lymph nodes for parasitic burden determination

**Timing: 3.5 h/16 mice**

Perform lymph node and ear harvesting immediately following euthanasia. In this section, we describe exclusively the process for lymph node collection.

4. 7 days post-infection, harvest the superficial parotid lymph nodes (Please refer to,^9^ page 311 diagram for proper identification of the superficial parotid lymph nodes).
  a. Secure mouse to a surgical wax tray using needles.
  b. Make a vertical incision from the top of the chest to the bottom of the mandibula.
  c. Peel back the neck muscle and tissue to expose the cervical lymph nodes on the side of infection.
    **CRITICAL:** Remove with autoclaved surgical forceps (curved forceps are optimal for removing small lymph nodes).
  d. Place the collected lymph node(s) in a 1.5 mL Eppendorf tube with 800 μL of parasite-specific media.
    ***Note:*** The lymph node(s) will sink in the media. As a reference, fat will float.
5. Add 3 mL of media to a sterile 50 mL falcon tube.
6. Place a 70 μM filter on top of the 50 mL falcon tube.
7. Decant the 1.5 mL Eppendorf tube containing lymph node(s) on to the 70 μM strainer.
8. Macerate the lymph node(s) against the cell strainer using the plunger end of a clean 3 mL syringe for 1 minute.
9. Wash the strainer and syringe plunger with 1 mL of media to retrieve all the cells into the suspension.
10. Centrifuge the cell suspension at 1800 rpm for 8 min.
11. Sample is now ready for plating for parasite burden estimation.

### Animal tissue harvesting and processing: Ears for parasitic burden determination

**Timing: 3 h/16 mice**

Harvest the ear tissue from mice and process the dermis to determine parasite burden at the site of intradermal infection.

12. Prepare a 6-well plate containing two wells each of 5 mL 70% ethanol, 5 mL PBS, and 5 mL DMEM medium.
13. Prepare a liberase solution (as described above) and add 1.5 mL to each well on a 6-well plate.
14. Remove ears with autoclaved surgical scissors and clean by dipping in each well listed in step 12 (70% ethanol first, PBS second, DMEM last).
15. Separate dermal layers on a sterile petri dish using two autoclaved surgical forceps.
16. Place the separated dermal layers facing downward in the 6-well plate containing the liberase solution.
17. Harvest all the ears and incubate at 37°C, 5% CO_2_ for 90 min.
18. Remove the ear from the plate with sterile surgical forceps and place in sterile medicon.
19. Place Medicon in a Medimachine to macerate the ear for 2-3 min.
20. While ear is macerating, label 15 mL Falcon tubes.
21. Place an 18-gauge needle attached to a 10 mL syringe into the medicon. Slowly draw up the macerated tissue (now a liquid homogenate) while simultaneously pipetting 10 mL of DMEM into the medicon with a 10 mL serological pipette.
22. Safely remove the needle from 10 mL syringe after drawing the entire sample into the syringe.
23. Place a Filcon cell filter on the opened, labeled 15 mL Falcon tube. Pass the sample from the syringe through the Filcon, discard the Filcon, and re-cap the tube.
24. Centrifuge at 1800 rpm for 8 min at 26°C.
25. Sample is now ready for same-day parasite burden plating.

### Parasite burden plate setup

**Timing: 2 h/16 mice**

Use the cell suspension obtained from the lymph nodes and ears to quantify the parasite burden by limiting dilution assay. Perform this process on the same day as the tissue processing.

26. Label a 96-well flat bottom plate with sample IDs.
27. Prepare 25 mL of parasite species-specific supplemented medium for parasite burden plating (see materials and equipment section) in a 25 mL sterile reservoir.
28. Using a multichannel pipette, add 150 μL of medium to each well.
29. Resuspend sample (pelleted ear homogenate or lymph node) in 150 μL of medium.
30. Add 150 μL of sample to the first well of the 96-well plate (Column 1, Row A; Column 1, Row B… etc.). One sample per row.
31. Using a multichannel pipette perform serial dilutions.
  a. Mix contents of Column 1 well by pipetting up and down.
  b. Remove 150 μL from Column 1 and transfer to Column 2
  c. Mix well by pipetting up and down.
  d. Transfer 150 μL to Column 3.
32. Repeat this process until Column 12. This will result in 1:2 serial dilutions across the plate.

***Note:*** Serial dilutions can be modified depending on the cell number and expected parasite count. If higher parasite loads are expected, perform 1:5 or 1:10 dilutions.

33. Discard the leftover 150 μL in the last well after performing dilutions to Column 12. At this point, each well should have a total volume of 150 μL.
34. Secure the lid to the plate by wrapping in parafilm.
35. Incubate at 26-27°C.
36. Every 3-4 days, top off each well with 100 μL of medium.
37. Reseal the plate with parafilm and observe under a microscope at 7- and 14-days post-plating.
38. Calculate log titer of parasites.
  a. When observing with an inverted microscope at 40X, record the last well where promastigotes are seen.
    ***Note:*** The number of the well (or the number of the dilution) will account for the logarithm exponent. The base will be 2 since the dilutions were performed 1:2 each time.
    ***Note:*** if the dilutions were not 1:2, but 1:10 you must use the dilution as the base of the logarithm.
  b. Graph in the Y axis the number of the well. The values reported in the graphs represent the highest log dilution with viable parasites.

### Animal tissue harvesting and processing: Ear tissue preparation for mass spectrometry

**Timing: 2 days (3 h sample preparation)**

Properly processing mouse ears for metabolomic analysis through mass spectrometry is crucial. It is important to keep the samples sterile to avoid contamination. Work quickly to preserve the metabolic profile after dissection.

39. After mouse euthanasia, collect infected ear and rinse it in 1X PBS.
40. Insert the ear in a screw cap sterile cryotube to avoid contamination.
41. Snap freeze the tube for 30 seconds in liquid nitrogen to quench.

**CRITICAL:** At this step, samples can be shipped in dry ice. Further processing was performed at the Metabolomics Core Facility.

42. Add 500 μL of 100% MeOH to the samples and sonicate in water-bath for 15 min to wash the tissue before homogenization.
43. Weigh the tissue and homogenize for 3 min at 40 mg/mL of 50% MeOH solution for 3 cycles in a Bertin Precellys Cryolys Evolution homogenizer using 2 mL soft tissue bead vials.
44. Centrifuge vials at RCF of 13500 x g for 15 min at room temperature (20-22°C).
45. Collect the supernatant above the protein precipitate containing metabolites for LC-MS.
46. Dry down the sample in a fume-hood on a hotplate (35°C) for approximately 30 min or until the sample is completely dry.
47. Reconstitute the pellet in ½ of the original volume in 5% MeOH with 0.1% formic acid. Add 250 μL into the tube and sonicate for 30 min to encourage the pellet to dissolve. Following this the tube will be centrifuged at 13500 x g to pull down any particulate remaining and place the supernatant into glass vials for LC-MS analysis.
48. Make pooled quality control samples are by aliquoting 10 μL of each sample into a single vial, so all groups are equally represented.
49. Run vials between technical replicate batches of experimental samples and blanks with three sets of replicates as we describe in the following sample sequence.

***Note:*** Blank1, Blank2, Blank3, Pooled-QC1, Sample1…SampleX, Blank4, Blank5, Pooled-QC2, Sample1-2…SampleX-2, Blank6, etc….

***Note:*** Sample sequences included all groups in the batch, which are staggered to avoid higher batch effects for particular groups over others in the sequence.

50. Perform untargeted analysis on a Thermo Orbitrap LTQ XL with HPLC separation on a Poroshell 120 SB-C18 (2 × 100 mm, 2.7 mm particle size) with a WPS 3000 LC system.
  a. Prepare a gradient consisting of solvent A, H_2_O with 0.1% Formic acid, and solvent B 100% acetonitrile.
  b. The gradient should be set up at 200 mL/min flow rate with an initial 2% solvent B with a linear ramp to 95% B at 15 min, holding at 95% B for 1 minute, and back to 2% B from 16 min and equilibration of 2% B until min 32.
  c. For each sample, inject 5 μL and select the top 5 ions for data dependent analysis with a 15 second exclusion window.
51. Use progenesis QI version 2.4 for feature selection in the untargeted results analysis, including database comparison and statistical processing where positive and negative mode runs are performed separately but in the exact same workflow (except for adduct selection).
  a. Upload Thermo .RAW files into the Progenesis QI in profile mode using a no import filter setting (0.0). Include all blank and pooled QC samples.
  b. Perform automatic processing and include all pooled QC samples as the alignment reference for all samples.
    ***Note:*** For peak picking settings choose a sensitivity level of 5 and adducts of [M]^+^, [M+H]^+^, [M+Na]^+^, [M+K]^+^, and [2M+H]^+^ for positive mode and [M]^-^, [M-H]^-^, and [M+Cl]^-^ for negative mode.
  c. A set of feature cut-offs were created and applied to find the most significant features for further investigation:
    i. Features that were highest in blank were tagged and removed.
      ***Note:*** This might retain those features that are derived from the background that are slightly higher in a sample group compared to the blanks, but the following QC filtering should also remove these features.
    ii. A minimum CV for pooled QC samples <30% was applied; remove those above this cutoff. As the pooled QC’s contained all features for each group, only apply QC CV% filtering.
    iii. ANOVA *p*-value scores between the groups with a cutoff of < 0.05 was applied. This was done for each group comparison as these samples were independent of each other (*i.e.* parallel sets of infections are compared to non-infected controls in each case).
      ***Note:*** Normality was also assumed based on the previous cut off for pooled QC of <30% and therefore ANOVA *p*-value was appropriate and only these features were used for further analysis to remove any possible metabolites not a part of the pathways altered via infection.
52. Perform a tentative identification of this set of filtered features using Progenesis MetaScope using the latest HMDB and LIPID MAPS databases at 10 ppm mass tolerance for MS and 20 ppm for MSMS.

***Note:*** Due to slightly lower resolution for the Orbitrap in MSMS mode to increase speed and the lower need for high mass accuracy in fragmentation pattern matching).

***Note:*** For complete validation of each detected metabolite, standards with matching retention times should also be used but in the scope of this study this was used to generate level 2 confidence of feature identification (accurate mass and MSMS library matching).

***Note:*** The output of the mass spectrometry will be two Excel files, pertaining to the positive and negative mode datasets, which contain the annotated metabolites and their respective abundances in each sample. Use the list of tentatively identified features for further validation and pathway analysis.

### Identification of significantly altered pathways in metabolomic datasets

**Timing: 4 h for setting up MetaboAnalyst + 2–4 weeks for further analysis**

The following section explains how to prepare the input files for the pathway and network analysis software.

***Note:*** It is preferable to use two independent approaches for the identification of the enriched or altered pathways in metabolomic datasets.

***Note:*** Using the Functional Analysis Module (MS peaks to pathways) in MetaboAnalyst 4.0 and the MetScape 3.1.3 algorithm allows for the identification of enriched pathways and for the construction of integrative networks to visualize the interaction of pathways with each other. An overall tutorial on how to use MetaboAnalyst is readily available on their website: https://www.metaboanalyst.ca/MetaboAnalyst/docs/Tutorials.xhtml

53. Remove metabolites with fragmentation scores of less than 20 from further analysis.
54. Calculate the fold change of each metabolite between the infection groups (i.e. *L. major* WT vs naïve), by dividing the mean concentration across the replicates for each group.
55. Using the Data Analysis toolkit on Excel, perform a Shapiro-Wilk test to assess whether the dataset is normally distributed.

***Note:*** A significant *p*-value of 0.05 or smaller signifies a non-parametric distribution. Determining that normality of a dataset before performing statistical significance testing is of paramount importance, because certain significance tests require a normally distributed dataset as their input. Determine which significance tests are the most appropriate to run by performing a normality test first.

***Note:*** Our dataset passed the Shapiro-Wilk test, and so we concluded that our dataset was normally distributed.

***Note:*** Since, according to the Shapiro-Wilk test, our dataset was normally distributed, we used a two-tailed student’s t-test as our test of statistical significance. Perform a Mann Whitney’s U test if the data had not been normally distributed.

56. Use Mann-Whitney test to calculate the *p*-value on the two samples with equal variance between the groups. Adjusted *p*-values are generated using multiple comparison testing correction to adjust for the appearance of false positives.

***Note:*** Bonferroni and the False Discovery Rate (FDR) are the two most commonly used methods of multiple correction testing used on large datasets, with the Bonferroni adjustment acting as the more conservative method by adjusting for the probability of identifying one false positive feature, while the FDR method controls the error rate among a set of tests. The calculated adjusted *p*-value should have a significance cutoff point of 0.05.^10^

57. Calculate the t-score according to the formula (x-u)/(S/sqrt(N)), in which x is the sample mean, u is the population mean, S is the standard deviation of the sample, and the sqrt(N) is the square root of the sample size.
58. Use the Functional Analysis Module (MS peaks to pathways) in MetaboAnalyst 6.0 to identify the significantly altered pathways and the functional differences between the infection groups.
  a. **CRITICAL:** MetaboAnalyst requires either a peak list profile or a peak intensity table as an input file to perform functional analysis on the dataset.

***Note:*** A peak list profile has 4 columns containing the mass-to-charge ratio (m/z), the retention time (rt), the *p*-value (24.c), and the t-score (24.d).

***Note:*** A peak Intensity table consists of the individual feature intensities and a column containing their respective mass-to-charge ratio/retention time.

***Note:*** Neither input file requires HMDB IDs, and both must be uploaded in Comma Separated Values (CSV) or Plain Text (txt) format.

59. Upload the peak list profile or peak intensity table to the functional analysis module. For our experiment, we used a peak list profile.

***Note:*** Examples of input files for MetaboAnalyst are available on their website (https://www.metaboanalyst.ca/MetaboAnalyst/upload/PeakUploadView.xhtml)

60. On the upload page, set the ion mode to positive, negative, or mixed depending on the analytical mode used to generate the data.

***Note:*** Information regarding the mass tolerance and the retention time units are available in the mass spectrometry output file and should be verified with the group that ran the mass spectrometry.

***Note:*** In our experiment, the mass tolerance is set to 10 ppm and the retention time units set to min.

61. Check the option to enforce primary ions.

***Note:*** This option is not available for the upload of peak intensity files.

62. Perform an internal data integrity check which will be considered passed if at least three replicates are present for each class of data, and the data does not contain non-numeric values. Remove and missing values at this step. Remove any missing values at this step.
63. In the parameter settings, use the Mummichog algorithm (version 2.0) and the Modified Gene Set Enrichment Algorithm (GSEA) for all analyses.
64. Leave the *p*-value cutoff for the mummichog algorithm at the default (top 10% of peaks).
65. Leave currency metabolites and adducts at default settings.

***Note:*** Depending on the host and pathogen of interest, select the appropriate pathway library that the functional analysis module will use to infer pathway activity.

***Note:*** We selected the Kyoto Encyclopedia of Genes and Genomes (KEGG) pathway library for *Mus musculus* to match our experimental model organism. Allow only pathways/ metabolite sets with at least three entries.

***Note:*** The output of MS peaks to pathways is a scatter graph in which each node on the graph represents a detected pathway, and the size of the node corresponds to the pathway’s enrichment. The graph is divided into four quadrants, each correlating to one or a combination of GSEA and mummichog algorithms. The quadrant at the upper right corner corresponds to pathways that were identified as enriched by both algorithms; therefore, they could potentially serve as a meaningful starting point for detailed analysis.

***Note:*** All pathways enrichment results are summarized in a provided table, and double clicking each node will provide more detailed information regarding the compound matching for the selected pathway.

66. Use the MetScape 3.1.3 App hosted on the Cytoscape software to build integrative network analysis.

***Note:*** Cytoscape 3.4.0 or higher is necessary to run the MetScape algorithm.

***Note:*** This analysis uses MetScape’s internal database which incorporates KEGG and EHMN data.

67. Convert the IDs of the metabolomic dataset from HMDB IDs to KEGG IDs recognized by the MetScape algorithm using the Chemical Translation Service (CTS) or the MetaboAnalyst Compound ID Conversion tool.
  ***Note:*** MetScape input files must contain metabolites identified with KEGG IDs and their corresponding fold change ratios and *p*-values for the comparison of interest between infection groups (i.e. *L. major* WT vs naïve).
  ***Note:*** Some HMDB IDs do not have corresponding KEGG IDs, remove these from this analysis.
  a. Select the option to build a pathway-based network.
    ***Note:*** MetScape can also build correlation-based networks. Input files for this form of network building can be made using the correlation calculator tool available on the MetScape homepage (http://metscape.ncibi.org/calculator.html).
  b. Select the organism of choice from among human, mouse, or rat.
  c. Upload the compound file with the updated KEGG IDs to MetScape.
    ***Note:*** MetScape focuses on human metabolic pathways and mostly covers endogenous primary metabolism. It is likely that it will not recognize several KEGG metabolites in the dataset being uploaded and will automatically remove them from further analysis.
  d. Set the *p*-value threshold at 0.05 and the FC Ratio threshold to 1.5.
  e. Create a compound-gene network.
    **CRITICAL:** Depending on the interactions of interest, you can create a compound-gene, compound-compound, or compound-reaction-enzyme-gene network for your dataset.
    ***Note:*** In our experiment, we created a compound-gene network, as we were interested in the impacted genes of our pathway for downstream in-vitro verification of our results via ELISA and PCR.
  f. Create a subnetwork of the metabolic pathway(s) of interest, in our case Tryptophan Metabolism and the pentose phosphate pathway, to allow the visualization of the integrated relationship among the metabolites and genes involved in this pathway(s).

### Metabolomic data analysis

**Timing: 2 h**

Statistical analysis of metabolic data, such as the generation of dimensionality reduction and volcano plots, are necessary for a thorough understanding of the structure of the dataset. These statistical methods allow us to visualize the potential inter-sample differences that exist in metabolite intensity across the dataset, which can consequently be explored in more detail via pathway and integrative network analyses. MetaboAnalyst offers statistical analysis modules, which can be used to generate volcano plots, heatmaps, and dimensionality reduction plots, such as Principal Component Analysis (PCA) and Partial Least Squares Discriminant Analysis (PLS-DA).

68. In the MetaboAnalyst software, format peak intensity data tables from the mass spectrometry experiment into comma-separated values (CSV) files conforming to MetaboAnalyst’s requirements.
  a. Upload the dataset to the appropriate statistical analysis module. In our experiment, we used the one factor statistical analysis.
    ***Note:*** Analyze the time series and multiple-factor datasets via the metadata table statistical analysis module.
  b. Perform an internal data integrity check, which will be considered passed if at least three replicates are present for each class of data, and the data does not contain non-numeric values. Any missing values will also be removed at this step.
  c. Perform an additional data filtering based on interquartile range.
  d. To perform data normalization, transformation, and scaling, navigate to the normalization overview page.
    ***Note:*** Normalization changes the distribution of the dataset, while scaling changes the range of the data and is helpful for use with distance-based models. Transformation is typically used with asymmetrical datasets and aims to correct the skewness of the data to reduce variability. Log-transformation is the most used method.
    ***Note:*** With each normalization, the results can be viewed in graph format. Performing normalization can be accomplished in many ways, and there is currently no consensus on which strategy to use. The best normalization procedures are dependent on the system and the dataset being studied.
    ***Note:*** Our data was autoscaled and normalized based on the median, but no transformation was performed.
    ***Note:*** Pre-processing of metabolomic data can generate datasets that are more compatible with MetaboAnalyst modeling.
  e. Apply dimensionality reduction, both principal component analysis (PCA) and partial least-squares discriminant analysis (PLS-DA).
    ***Note:*** Using MetaboAnalyst, perform a quality analysis of the PCA and PLS-DA using the pooled QC. If these are clustered together this will provide quality assurance of the analysis.
  f. Use the cross-validated sum of squares (Q2) performance measures to determine if PLS-DA models were overfitted. Visualize the significant, differentially regulated metabolites by generating volcano plots with cutoffs of <0.05 false-discovery rate (FDR) and >2-fold change (FC).
    ***Note:*** 2-fold change is the default setting on MetaboAnalyst; however, the fold change can be reduced if the user wishes.
  g. Create dendrograms to analyze clustering of samples.
  h. Create hierarchical heatmaps to analyze features.

### Identification of relevant metabolites

In this section, we describe our logic in identifying metabolic pathways of interest. Our process drew upon literature review as opposed to a stepwise protocol. Rather than a step-by-step protocol, this was mostly done based on literature research.

**CRITICAL:** Until this step, differentially abundant metabolites among groups are clear.

Nevertheless, this information alone is not sufficient to determine which metabolites are involved in mediating protection against infection or in responding to other scientific questions. In our opinion it is crucial to investigate the interactions between these metabolites and conduct pathway analysis to gain a more complete understanding of the biological mechanisms at play. For example, we might see a significant increase in Metabolite A in one of the groups. However, if Metabolite B from the same pathway is simultaneously decreased, there will be no net change in the pathway dynamics between the groups, making this pathway biologically irrelevant. This is why in addition to the up/downregulation of single metabolites; it is crucial to determine whether the pathway is overall de/activated. Focusing on the scientific question to determine which pathways might be the most interesting in the context of the disease/condition under study is critical. In our case, the pentose phosphate pathway (PPP) was differentially expressed in *L. mexicana* WT vs *L. mexicana Cen*^*-/*^ *(LmexCen*^−/−^*)*^*-*^. PPP promotes M1 polarization in macrophages, suggesting a switch to a pro-inflammatory phenotype following *LmexCen*^−/−^ inoculation (Volpedo et. al. iScience 2023). Conversely, in the case of *L. major* WT and *L. major Cen*^−/−^ we identified tryptophan metabolism as the most significant difference which had a role in the immune response (Oljuskin et. al. iScience 2023). As an example, we have used a similar methodological approach to study the pain-reducing metabolomic reprogramming in *L. mexicana* WT infection. In this case, we focused on pathways already known to be involved in analgesia.^11^ We strongly recommend the researchers to rely not only on the metabolites list but also on the literature to choose the pathway that responds to their specific research question and always consider the biological relevance of your study.

### *In vitro* validation: Generation of BMDMs/BMDCs

**Timing: 5 days**

Perform *in vitro* validation by administering an inhibitory drug targeting the metabolic pathway identified. We recommend to carefully select the cell type of interest. We have chosen macrophages and dendritic cells (DCs) for our validation experiments since phagocytes are the main hosts for *Leishmania* parasites.

69. Collect femurs from both hind legs of naïve mice to recover bone marrow aspirates for BMDM or BMDC infections.
  a. Isolate the femur and tibias from C57BL/6 mice in sterile conditions.
    ***Note:*** Remove the leg by cutting above the femur to ensure collection of the entire bone.
  b. Remove skin and muscle tissues around the bones using a scalpel and place the cleaned bones in complete RPMI.
  c. Cut the edges of the bones and keep them in complete RPMI until use.
70. Place the bones in a sterile petri dish.
71. Cut the distal and proximal epiphyses of the femur with a scalpel to expose bone marrow and flush.
  a. With a 25-gauge needle attached to a 3 mL syringe, flush the bone marrow with complete RPMI directly into 50 mL tubes containing 25 mL of complete RPMI.

***Note:*** Repeat the flushing until all bone marrow is collected into the tube, and the interior of the bone is visually cleared/translucent.

72. Centrifuge at 1600 rpm for 6 min at 4°C to pellet the cells. Discard the supernatant.
73. Add RBC Lysis buffer in an equivalent volume to the pellet.
74. Gently swirl the tube for 30 seconds
75. Stop the reaction by adding 10 ml of serum-free ice-cold RPMI.
76. Centrifuge at 1600 rpm for 6 min at 4°C to pellet the cells. Discard the supernatant.
77. Resuspend the cells in RPMI and count using the standard method of your lab.
78. Plate bone marrow cells and treat them with growth factors to differentiate them:
  a. For bone marrow derived dendritic cells (BMDCs,) plate 2 × 10^6^ cells per well in a 6-well plate. Add 150 μL GM-CSF and IL-4 to each well.
  b. For bone marrow derived macrophages (BMDMs) plate 0.5 × 10^6^ cells per well in a 24 well plate. Resuspend cells at 1 × 10^6^ cells/mL in complete RPMI containing 20 ng/mL of murine M-CSF.

***Alternatives:*** BMDMs can also be differentiated by using complete RPMI containing 20% supernatant from L-929 cell line

79. Incubate the plates at 37°C with 5% CO_2_ for 4-7 days until cell differentiation is complete.
  ***Note:*** Differentiation is considered complete when:
  a. Cells have adhered to the bottom of the plate.
  b. Cells have a star-like and elongated shape rather than rounded.
  c. Cells DO NOT present many vesicles in their cytoplasm. The presence of vesicles (observed as dots and empty circles in the cells) are a sign of an old culture with cells that are no longer viable for experiments.
80. Routinely aspirate medium (approximately every 3 days) to remove non-adherent cells and provide fresh medium and growth factors to cell cultures.

### *In vitro* infection of BMDCs/BMDMs and pathway inhibition

**Timing: 3 days**

Here, we present the steps to an *in vitro model* of infection of target cells by *Leishmania* parasites, which allows the downstream evaluation of immune response by cytokine production when using drugs that alter the targeted signaling pathways.

***Note:*** In our case, since the pentose phosphate pathway (enriched in *L. mexicana Cen*^−/−^ infection) activation results in inflammatory cytokine production, measuring IL-12 and IL-1β via ELISA is a common method to validate the experiment. Adapting different inhibitors and cytokines is possible with this method based on enriched pathways with different infections.

We used a different validation approach for the *L. major Cen*^−/−^, which immunization results in the alteration of tryptophan metabolism towards the increased production of melatonin and the inhibition of kynurenine and 6-formylindolo [3,2-b] carbazole (FICZ). As enrichment of melatonin leads to an induction of pro-inflammatory cytokines, such as TNF-α and IL-12, we measured the transcripts level of these cytokines via RT-PCR in the presence of melatonin and the melatonin inhibitor.

**CRITICAL:** The best assay to perform will depend on the differently expressed pathways and their role when active/inactive, (i.e., phagocytosis assay, cell morphology evaluation, surface markers expression, *etc.*).

81. Add parasites to the cell culture with an IMO of 10. That is 10 times more the number of plated cells per well.

***Note:*** Do not forget to include non-infected controls.

82. Incubate for 18 h at 37°C with 5% CO_2_.
83. Remove the extracellular parasites by washing the cells twice with warmed (37°C) complete RPMI.
84. Add LPS at a final concentration of 0.75 μg/mL in warmed complete RPMI.
85. Incubate for 24 h at 37°C with 5% CO_2_.
86. Add the appropriate inhibitors and collect the sample.
  a. For BMDMs: Add the PPP inhibitors: 6-aminonicotinamide (6-AN) or dehydroepiandrosterone (DHEA) (at a final 100 μM final concentration in complete RPMI.
  b. For BMDCs: Add the treatment of choice at the following concentrations:
    i. 600 μM of 1-methyl tryptophan (L-isomer)
    ii. 50 μM of kynurenine (L-isomer)
    iii. 50 ng/mL of melatonin
    iv. 10 μM of 4-CDP
    v. 5 mM of FICZ
87. Incubate the plate with the treatment for 24 h at 37C.
88. Recover the sample to perform further analysis.
  a. For BMDMs: Recover supernatant for use in cytokine ELISA assays.
  b. For BMDCs: Scrape the adhered cells from the wells for RNA extraction and RT-PCR for the indicated genes.

### Preparation of samples for RT-PCR

**Timing: 6 h/16 mice**

Here, we describe the process to synthesize cDNA from the RNA extracted from the cells. Then we present the process to perform a PCR with such cDNA.

89. Extract RNA from bone marrow-derived cells by first scraping the wells.
  a. Aspirate medium using a vacuum waste system.
  b. Add 1.5 mL sterile PBS to each well.
    ***Note:*** If pooling two or more replicates together into one sample, ensure that the final PBS volume used among them is 1.5 mL
  c. Using one cell scraper per well, scrape cells for approximately 2 min until sample is slightly opaque.
    ***Note:*** At this step, confirm the removal of adhered cells from the plate by observing the plate under a microscope.
  d. Add sample to a labeled 1.5–2 mL tube and centrifuge at 1500 rpm for 10 min.
90. Complete an RNA extraction by following the Invitrogen PureLink RNA Mini Kit protocol.
91. Measure the RNA yield of each sample using a spectrophotometer.
92. Using the sample with the lowest RNA yield, standardize the RNA concentrations to generate cDNA.

***Note:*** The lowest amount of RNA used to successfully synthesize cDNA was 3 ng/μL, however higher concentrations may provide more accurate results.

93. Prepare cDNA in the thermocycler using the High-Capacity cDNA Reverse Transcription Kit following manufacturer’s instructions.

**Pause Point:** Store cDNA at −80°C for 2 up to weeks.

94. Prepare RT-PCR master mix including TaqMan Gene Expression Assay, specific RT-PCR probes, nuclease-free H_2_O, and 5 μL of cDNA sample.
95. Plate on a clear, 96-well PCR plate. Cover with PCR film and spin at 2000 rpm for 5 min.
96. Run RT-PCR using the following cycling conditions.

**Table:** PCR cycling conditions

Steps	Temperature	Time	Cycle
Initial Denaturation	50 C	2 mins	1
Denaturation	95 C	10 mins	1
Annealing	95 C	0:15 mins	39
Extension	60 C	1 min	
Hold	4 C	30 mins	1

97. Calculate gene expression relative to a housekeeping gene.

### *In vitro* validation via ELISA

**Timing: 3 days**

In this section, we describe the protocol for sandwich ELISA to identify the cytokines released to the supernatant by the infected cells after in vitro infection.

98. Perform sandwich ELISA using the appropriate antibodies, IL-12 and IL-1β are shown in this protocol:
  a. Coat a high binding 96 well plate with 2 μg/mL of capture antibody in 1X PBS at pH 9 using 50 μl per well.
  b. Incubate overnight (18-20 h) at 4C wrapped in plastic foil.
    **CRITICAL:** Do not stack the plates on top of each other as this could cause “plate effect” and lead to different binding among the wells.
  c. Bring the plates to room temperature (20-22°C) for about 20-30 min.
  d. Flick the plate to remove the capture antibody.
    **CRITICAL:** Do not allow the plate to dry completely during the assay.
  e. Add 200 μl of room temperature (20-22°C) blocking buffer (10% FBS in PBS) to each well.
  f. Incubate for 2 h at room temperature (20-22°C) covered with plastic foil.
  g. Wash the plate in PBS and PBS + Tween.
  h. Prepare a Standard curve by using a recombinant protein serial dilution in blocking buffer. Although this step varies among cytokines and samples, we recommend a starting concentration of 10 ng/mL with 1:2 serial dilutions.
  i. Add 50 μl of sample to each well.
    ***Note:*** We recommend using technical duplicates for each sample.
  j. Wrap the plates in plastic and incubate at 4C overnight (18-20 h).
99. Develop the ELISA using colorimetric reagents such as TMB.
  a. Bring the plates to room temperature (20-22°C) before starting.
  b. Wash the plate twice in PBS and PBS + Tween.
  c. Add 50 μl of a biotinylated antibody to a concentration of 1 μg/mL in Blocking buffer.
    **CRITICAL:** Use an antibody with a different clone number than the capture antibody. This will allow both antibodies to bind to different domains of the protein. Using the same clones can result in the underestimation or lack of detection of the protein.
  d. Incubate at room temperature (20-22°C) for 1 hr.
  e. Wash the plate in PBS and PBS + Tween.
  f. Add 50 μl of Avidin D-HRP solution (5 μg/mL).
  g. Incubate 30 min at room temperature (20-22°C).
  h. Wash the plate in PBS and PBS + Tween.
  i. Add 100 μl/well of TMB solution (prepared by mixing equal volumes of Solution A and B from TMB peroxidase kit)
  j. Wait until the plate changes color.
    **CRITICAL:** Developing time varies among cytokines. Use the same incubation time for the same cytokine among different plates. To determine the optimal point to stop the reaction, be sure to meet the following criteria:
    i. The standard curve wells display a gradient color blue. Overprocessing of the plate results in similar intensity of the color throughout the standard curve wells.
    ii. The blank wells are completely transparent. Overprocessing of the plate will result in blue colored blank wells.
    iii. The samples developed a color that can be visually interpolated within the standard curve.
  k. Stop the reaction adding 50 μl of 5% Phosphoric acid to each well.
100. Read the plate at λ=450 nm.

## Expected outcomes

The primary data sets expected to be produced from this work include a variety of formats. For the mass spectrometry experiments, Excel files with m/z ratios, and the metabolite identifications should be generated. CSV input files will be needed for the Metaboanalyst analysis. Additionally, Metaboanalyst will generate the GSEA vs Mummichog pathway analysis graphs, and the ID conversion output file from either the Metaboanalyst ID tool, or the Chemical Translation Service. For Metscape, use Excel input files to generate the integrative network plots.

## Limitations

The results show one of the complications of untargeted metabolomics, which is the presence of multiple isoforms of the same compound that can sometimes have opposite fold change directions and create potential ambiguity in the interpretation of the results. Additionally, the annotation of the metabolites is tentative as they are based exclusively on the formula with no additional information regarding the compound structures. Another limitation is that metabolite identification is relies on m/z ratios and can sometimes lead to the misidentification of metabolites. For instance, a compound that should not be possible to find naturally in mammalian cells, such as caffeine, might be incorrectly identified. It is important for the researcher to keep in mind biological feasibility and relevance when analyzing the data in order to recognize and remove misidentified compounds from the analysis. Furthermore, it is important to keep in mind that some identified metabolites could have their origin in the infecting parasites, rather than the host cells themselves. Since the control samples lack any parasite metabolite, misinterpretations could occur. Researchers should consider all such information to determine the pathways chosen for the validation studies.

## Troubleshooting

### Problem 1

*Leishmania* cultures growing too slow or not growing (related to step 6 from Generating virulent parasite stocks section and to the section Reviving parasite cultures).

### Potential solution

• Confirm that the FBS used has been properly heat inactivated.
• Add 2 μg/mL of biopterin to the media when thawing the parasites or when recovering amastigotes.

### Problem 2

After tissue homogenization with the medicon machine [related to step 19 from animal tissue harvesting and processing: ears for parasitic burden determination section], the tissue is visibly not digested.

### Potential solution

Extend the tissue digestion for 2-3 additional min. Make sure to not remove or add any more liquid from the medicon unit. With sterile forceps, try to push the remaining tissue under the blade to ensure a more complete homogenization.

### Problem 3

After LC-MS metabolomics, statistical analysis of data does not show a reasonable statistical differentiation of the groups.

### Potential solutions

There are a number of possibilities when not observing a clear discrimination of the groups in the experimental study. A different combination of data normalization, scaling, and transformation might be needed before dimensionality reduction and statistical analysis is completed. If dimensionality reduction does not show a differentiation of groups, it is possible that the algorithm used is not well-suited to the dataset, and that a different algorithm may be a better fit.

## Resource availability

### Lead contact

Further information and requests for resources and reagents should be directed to and will be fulfilled by the lead contact, Dr. Sreenivas Gannavaram (Sreenivas.gannavaram@fda.hhs.gov).

### Technical contact

Further technical information should be directed to the technical contact, Dr. Sreenivas Gannavaram (Sreenivas.gannavaram@fda.hhs.gov).

### Materials availability

These studies did not generate new unique reagents. Any additional request should be submitted to the lead author for consideration.

### Data and code availability

All relevant data is available in the main text and supplemental information. Any additional information can be provided upon reasonable request to the authors. This paper does not report original code. Any additional information required to reanalyze the data reported in this paper is available from the lead contact upon request.

## Acknowledgments

The study was funded by the Global Health Innovative Technology Fund to A.R.S. and S.H. (GHIT 2020-0134).

Our contributions are an informal communication and represent our own best judgment. These comments do not bind or obligate the FDA.

## Author contributions

T.P.-F. and L.K. drafted the manuscript. N.A., H.M., M.B., and G.V. revised the manuscript and created the figures. T.O., P.B., S.H., G.M., S.G., A.R.S., and H.L.N. reviewed the manuscript.

## Declaration of interests

The FDA is currently a co-owner of two US patents that claim attenuated *Leishmania* species with the *centrin* gene deletion (US7,887,812 and US8,877,213). This article reflects the views of the authors and should not be construed to represent FDA’s views or policies.
